# Epigenome-wide association studies of meat traits in Chinese Yorkshire pigs highlights several DNA methylation loci and genes

**DOI:** 10.3389/fgene.2022.1028711

**Published:** 2023-01-04

**Authors:** Kai Wang, Shujie Wang, Xiang Ji, Dong Chen, Qi Shen, Yang Yu, Pingxian Wu, Xuewei Li, Guoqing Tang

**Affiliations:** ^1^ Farm Animal Genetic Resources Exploration and Innovation Key Laboratory of Sichuan Province, Sichuan Agricultural University, Chengdu, China; ^2^ Chongqing Academy of Animal Science, Chongqing, China

**Keywords:** DNA methylation, EWAS, CPGs, pig, gene

## Abstract

In this study, we aimed to identified CpG sites at which DNA methylation levels are associated with meat quality traits in 140 Yorkshire pigs, including pH at 45 min (pH_45min_), pH at 24 h (pH_24h_), drip loss (DL), meat redness value (a*), yellowness (b*) and lightness (L*). Genome-wide methylation levels were measured in muscular tissue using reduced representation bisulfite sequencing (RRBS). Associations between DNA methylation levels and meat quality traits were examined using linear mixed-effect models that were adjusted for gender, year, month and body weight. A Bonferroni-corrected *p*-value lower than $7.79\times 10^{-8}$ was considered statistically significant threshold. Eight CpG sites were associated with DL, including CpG sites annotated to *RBM4* gene (cpg301054, cpg301055, cpg301058, cpg301059, cpg301066, cpg301072 and cpg301073) and *NCAM1* gene (cpg1802985). Two CpG sites were associated with b*, including *RNFT1* and *MED13* (cpg2272837) and *TRIM37* gene (cpg2270611). Five CpG sites were associated with L*, including *GSDMA* and *LRRC3C* gene (cpg2252750) and ENSSSCG00000043539 and *IRX1* gene (cpg2820178, cpg2820179, cpg2820181 and cpg2820182). No significant associations were observed with pH_45min_, pH_24h_ or a*. We reported associations of meat quality traits with DNA methylation and identified some candidate genes associated with these traits, such as *NCAM1, MED13 and TRIM37* gene. These results provide new insight into the epigenetic molecular mechanisms of meat quality traits in pigs.

## 1 Introduction

Meat quality are important traits in the pig industry. Meat quality traits mainly include, pH_45m_, pH_24h_, water-holding capacity (WHC) or DL, meat color and intramuscular fat content (IMF). In the process of pig genetics and breeding, the production performance of pig has always been the main breeding goal, and has achieved remarkable results. However, in recent years, the meat quality of pig has attracted more and more attention. Improving meat quality has become a high priority for the pork industry.

A great deal of progress has been made by genome-wide association studies (GWAS) to identify genetic loci for meat quality traits (Zhang et al., 2015; Shen et al., 2016; Verardo et al., 2017; Xing et al., 2019). In recent decades, more than 30,000 quantitative trait loci (QTL) have been released for public access on the Pig QTLdb (release 40 December 2019. http://www.animalgenome.org/cgi-bin/QTLdb/SS/index). Among them, 730 QTLs have been found to affect pork pH and 651 QTLs are associated with meat color.

While tremendous progress has been made in identifying QTLs associated with meat quality traits, epigenetic mechanisms for regulating gene expression are less understood, such as DNA methylation, histone modification and chromatin accessibility. In particular, DNA methylation at CpG sites plays an important role in development, cell differentiation, imprinting and regulation of gene expression. DNA methylation is an annotation system that marks genetic text to guide how and when to read information and control transcription (Dor and Cedar, 2018). DNA methylation has been shown to be related to pig traits, including growth (Jin et al., 2014), reproduction (Bell et al., 2011) and immune response (Wang et al., 2017).

Similar to GWAS, epigenome-wide association studies (EWAS) use epigenetic factors instead of SNP to identify candidate genes for traits (Flanagan, 2015). In recent years, EWAS have identified associations for DNA methylation and complex traits in humans, such as body-mass index (BMI) (Dick et al., 2014; Demerath et al., 2015), obesity (Silva and Garvin, 2009; Klodian et al., 2018) and diseases (Dedeurwaerder et al., 2011; Mathias et al., 2016; Soriano-Tárraga et al., 2016; Meeks et al., 2019). However, up to now, most EWAS studies have been carried out in human but no EWAS studies have been conducted on pigs.

In this study, we aimed to investigate the association between DNA methylation and meat quality traits in Yorkshire pigs by using muscular tissue. We conducted EWAS using RRBS data and then identified 20 significant CpG sites associations with meat quality traits. The results are a step toward realizing the epigenetic molecular mechanisms of meat quality traits and identifying new loci.

## 2 Results

### 2.1 Animals and meat quality traits

A total of 140 Yorkshire pigs (51 male and 89 female) were sampled in this study. The characteristics of these pigs were presented in Table 1. The mean value of pH_45min_, pH_24h_, DL, a*, b*, and L* were 6.30, 5.91, 2.89%, 5.09, 2.36, and 45.77, respectively.

**TABLE 1:** Descriptive statistics of meat quality characteristics of muscular tissue from Yorkshire pigs.

Characteristics	Yorkshire pigs (mean ± ^a^SD)
N	140
Gender (male)	51
Weight (kg)	111.71 ± 12.97
pH_45_ _min_ ^b^	6.30 ± .29
^c^pH_24h_	5.91 ±.33
DL (%)^d^	2.89 ±3.14
a*^e^	5.09 ± 1.24
b*^f^	2.36 ± 3.66
L*^g^	45.77 ± 2.43

^a^SD, standard deviation.

^b^pH_45min_, pH at 45 min.

^c^ pH_24 h_, pH at 24 h.

^d^ DL, drip loss.

^e^ a*, meat redness value.

^f^ b, meat yellowness value.

^g^ L*, meat lightness value.

### 2.2 DNA methylation

**FIGURE 1:**
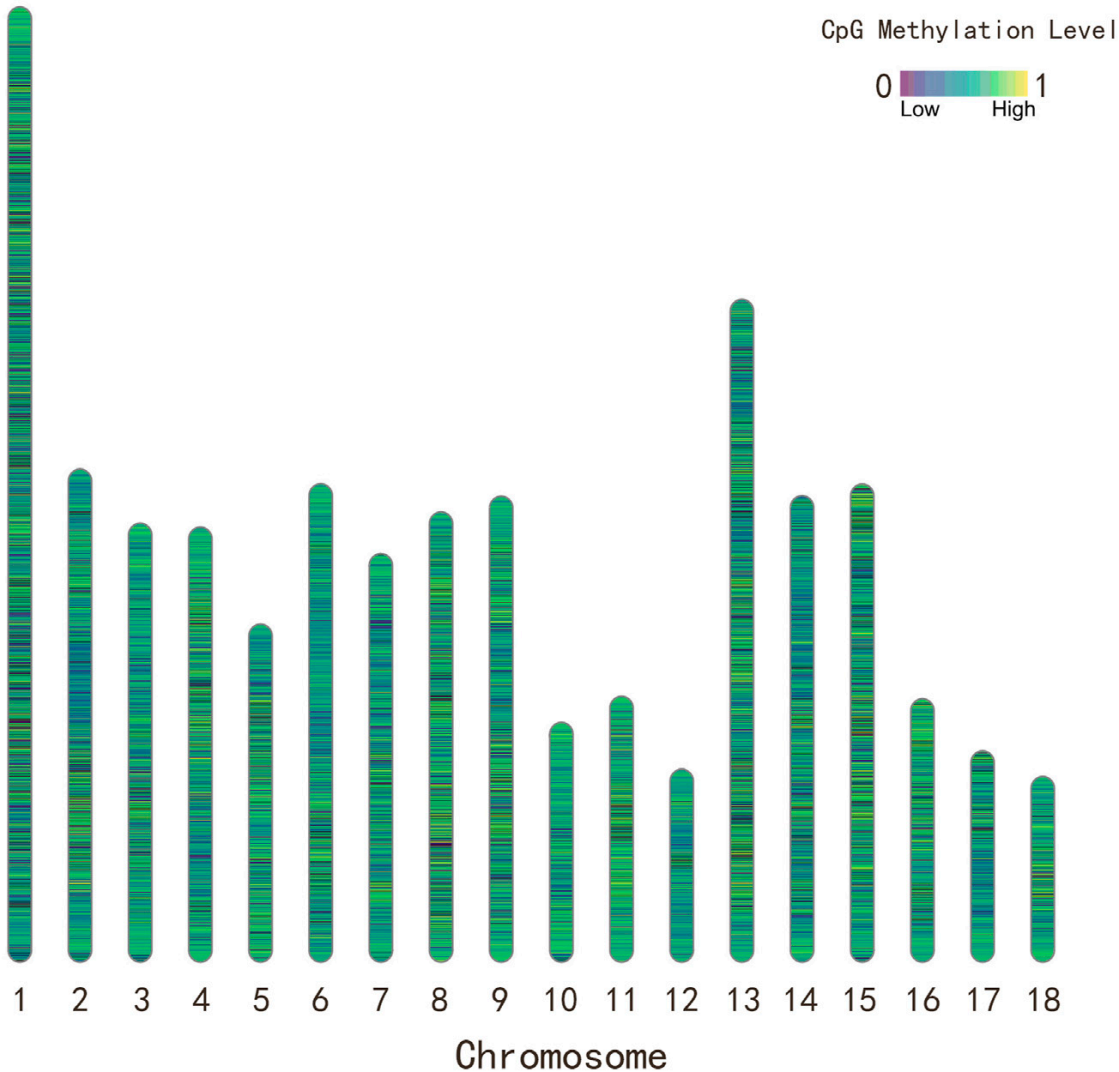
The distribution and methylation level of CpG sites for Yorkshire pigs in the 18 autosomal chromosomes. The color of chromosome ideogram represents the methylation level of CpG sites.

We constructed RRBS libraries from muscular tissue to examine methylation patterns in 140 pigs. We sequencing the libraries using Illumina HiSeq platform and then obtained on an average of 14.22±1.93 Gb raw bases per sample (Supplementary Table S1). After quality control, we obtained on an average of 11.28±1.74 Gb clean bases per sample. Moreover, the average bisulfite conversion of Yorkshire pigs was over 99%. Besides, more than 60% of the reads of Yorkshire pigs were mapped to the porcine reference genome. We filtered ML data for CpG sites with at least 10 × coverage, and present in at least 105 samples, corresponding to 3,083,713 CpG sites for further analysis. Figure 1 showed the distribution of CpG sites and methylation level of CpG sites for Yorkshire pigs in the 18 autosomal chromosomes.

### 2.3 EWAS

We performed EWAS between CpG ML and 6 meat quality metrics, including pH_45min_, pH_24h_, DL, a*, b* and L*. We then used the R package “CpGassoc” to determine associations between DNA methylation and phenotype, as is common practice for GWAS of quantitative traits. Based on the Bonferroni correction for the number of CpG sites tested, associations were deemed significant if *p*-value were below $7.79\times 10^{-8}$ .

**FIGURE 2:**
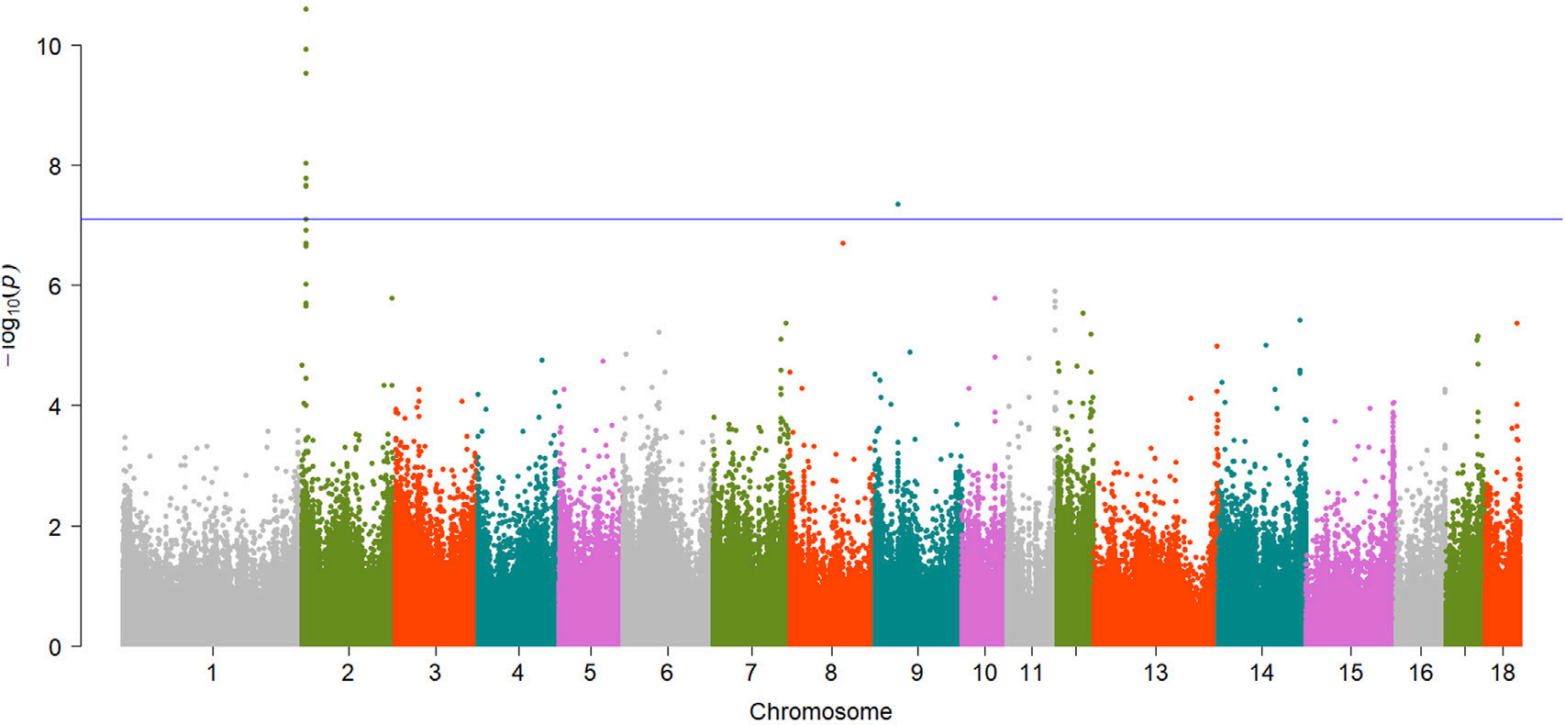
Manhattan plot showing EWAS results for DL trait. Each dot represents an CpG site and the blue line represents the threshold.

**FIGURE 3:**
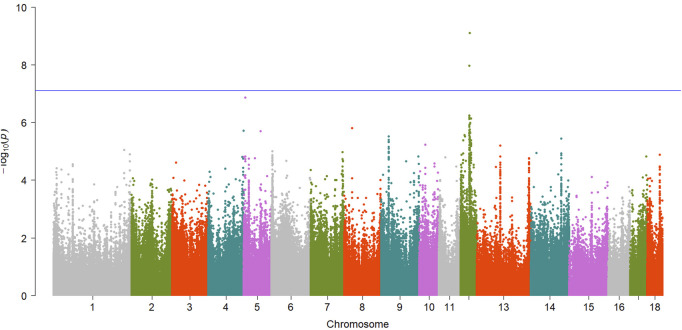
Manhattan plot showing EWAS results for b* trait. Each dot represents an CpG site and the blue line represents the threshold.

**FIGURE 4:**
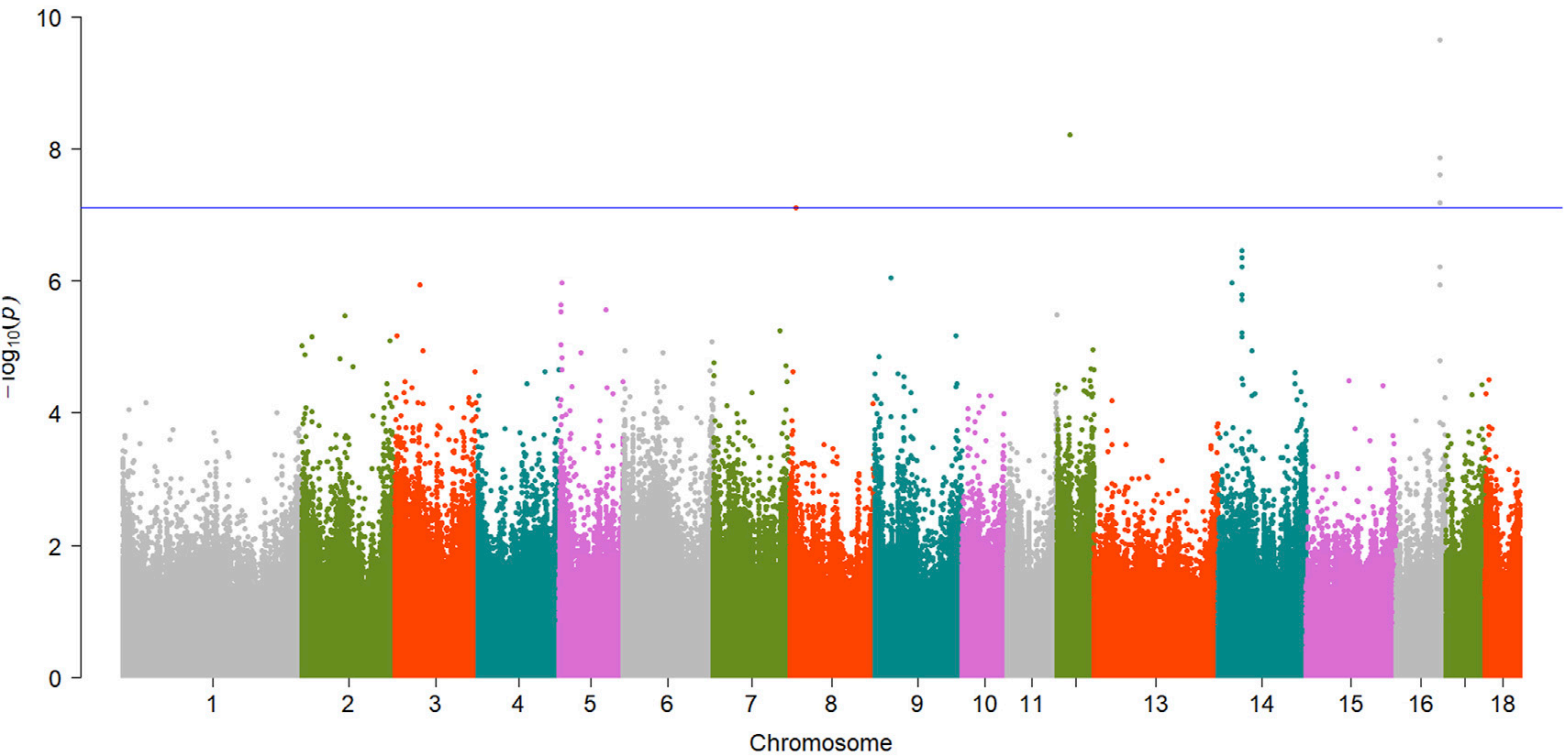
Manhattan plot showing EWAS results for L* trait. Each dot represents an CpG site and the blue line represents the threshold.

**FIGURE 5:**
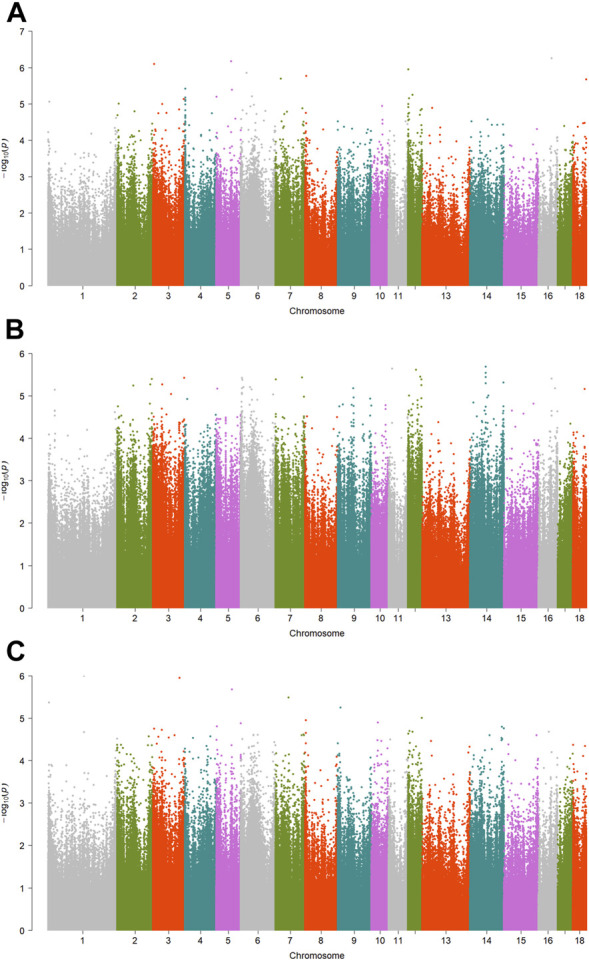
Manhattan plots showing EWAS results for other traits. Each dot represents an CpG site. **(A)** Manhattan plot showing EWAS results for pH_45min_. **(B)** Manhattan plot showing EWAS results for pH_24h_. **(C)** Manhattan plot showing EWAS results for a*.

A total of 15 significant associations were detected, corresponding to 3 unique phenotypes (Figure 2, Figure 3 and Figure 4) where the *p*-value was below $7.79\times 10^{-8}$ . Table 2 summarized the significant CpG sites associated with these traits. However, we did not find significant CpG sites ( $P<7.79\times 10^{-8}$ ) for pH_45min_ (Figure 5A), pH_24h_ (Figure 5B) and a* (Figure 5C).

**TABLE 2:** Summary of significant CpG sites associated with meat quality traits.

Traits	CpG sites	Chr	Pos	Intra/intergenic	Candidate genes	*p*-value
DL	cpg301073	2	5783171	Intragenic, exon	*RBM4*	2.47e-11
DL	cpg301059	2	5783071	Intragenic, exon	*RBM4*	1.13e-10
DL	cpg301055	2	5783024	Intragenic, exon	*RBM4*	2.85e-10
DL	cpg301058	2	5783070	Intragenic, exon	*RBM4*	9.17e-09
DL	cpg301066	2	5783114	Intragenic, exon	*RBM4*	1.63e-08
DL	cpg301054	2	5783023	Intragenic, exon	*RBM4*	2.14e-08
DL	cpg301072	2	5783170	Intragenic, exon	*RBM4*	2.20e-08
DL	cpg1802985	9	40844891	Intragenic, Intron	*NCAM1*	4.40e-08
b*	cpg2272837	12	36261646	Intergenic, 86,511 bp, 72,095 bp	*RNFT1*	7.72e-10
					*MED13*	
b*	cpg2270611	12	35340816	Intragenic, Intron	*TRIM37*	1.06e-08
L*	cpg2252750	12	22395823	Intergenic16,700 bp2,640 bp	*GSDMA*	6.05e-09
					*LRRC3C*	
L*	cpg2820181	16	77445768	Intergenic, 216,225 bp, 249,936 bp	ENSSSCG00000043539	2.20e-10
					*IRX1*	
L*	cpg2820178	16	77445749	Intergenic, 216,206 bp, 249,955 bp	ENSSSCG00000043539	1.3e-08
					*IRX1*	
L*	cpg2820182	16	77445775	Intergenic, 216,232 bp, 249,929 bp	ENSSSCG00000043539	2.44e-08
					*IRX1*	
L*	cpg2820179	16	77445750	Intergenic, 216,207 bp, 249,954 bp	ENSSSCG00000043539	6.61e-08
					*IRX1*	

As Table 2 shown, of the 15 significant CpG sites, 9 CpG sites were intragenic, and 6 CpG sites were intergenic. The distance between intergenic loci and nearby flanking genes ranged between 2,640 bp and 249,955 bp. The candidate genes listed for each site correspond to the gene itself for intragenic, and the two nearest flanking genes by distance for intergenic, with the distance between the site and each flanking gene listed for intergenic associations.

Subsequently, we calculated the average methylation level (ML) of significant CpG sites (Supplementary Table S2). Of these 15 significant CpG sites, 13 CpG sites (cpg301073, cpg301059, cpg301055, cpg301058, cpg301066, cpg301054, cpg301072, cpg2272837, cpg2270611, cpg2820181, cpg2820178, cpg2820182, and cpg2820179) were hypermethylation, and only 2 CpG sites (cpg1802985 and cpg2252750) were hypomethylation.

### 2.4 Candidate genes

Totals of 8 CpG sites reached significant level and were regarded as significant sites for DL trait (Table 2). Of the 8 CpG sites, 7 sites were located within the exon1 of *RBM4* gene (Figure S1), and the top CpG site was cpg301073 (SSC2:5783171, $P=2.47\times 10^{-11}$ ). Besides, only 1 CpG site (cpg1802985, SSC9:40844891, $P=4.40\times 10^{-8}$ ) were located within the *NCAM1* gene (Supplementary Figure S2).

For meat color trait, totals of 2 and 5 CpG sites reached significant level for b* and L*, respectively. For b*, one of them, cpg2272837 (SSC12:36261646, $P=7.72\times 10^{-10}$ ), was located in the intergenic region and the nearest flanking genes were *RNFT1* gene and *MED13* gene (Supplementary Figure S3). Another one was cpg2270611 (SSC12:35240816, $P=1.06\times 10^{-8}$ ) that was located within the *TRIM37* gene (Supplementary Figure S3). In addition, for L*, the nearest flanking genes of the cpg2252750 (SSC12:22395823, $P=6.05\times 10^{-9}$ ) were *GSDMA* and *LRRC3C* (Supplementary Figure S4). A total of 4 CpG sites were located in the intergenic region and the nearest flanking genes were ENSSSCG00000043539 and *IRX1* gene (Supplementary Figure S5). Of them, the top CpG site was cpg2820181 (SSC16:77445768, $P=2.20\times 10^{-10}$ ).

The main GO terms enriched in 9 candidate genes (Supplementary Table S1) might be related to negative regulation of centriole replication ( $P=2.4\times 10^{-3}$ ) and protein autoubiquitination ( $P=2.4\times 10^{-2}$ ).

## 3 Discussion

This study reported the results of EWAS of meat quality traits in 140 Yorkshire pigs, including drip loss (DL), meat pH (pH_45 min_, pH_24 h_), and meat color (a*, b* and L*). Just as genome-wide association studies (GWAS) grew from the field of genetic epidemiology, so too do EWAS derive from the field of epigenetic epidemiology. Although there have been numerous studies of EWAS in humans, few studies perform EWAS analysis to identified associations between DNA methylation and complex traits in livestock (Flanagan, 2015). In the current study, we found associations between DNA methylation levels and meat quality traits in the muscle tissue of pigs. These findings suggest that it was possible to find significant associations between DNA methylation and traits by using tissues associated with traits of interest. Besides, one of the advantages of using mammalian models, such as pigs, is that tissues that are not readily available in human studies can be collected.

DL trait is used to describe the water-holding capacity of meats. The water-holding capacity of meats is affected by multiple factors (Huff-Lonergan and Lonergan, 2005), including muscle cell structure, muscle contraction, muscle cell protein and genetic factors. We observed that methylation of 7 CpG sites annotated to the *RBM4* gene on SSC2 and 1 CpG site annotated to the *NCAM1* gene on SSC9 were associated with DL trait. The *RBM4* gene encodes RNA Binding Motif Protein 4 (RBM4) which participates in both precursor mRNA splicing regulation and translational control in muscle cells. RBM4 protein promotes the expression of many muscle-specific mRNAs from individual genes *via* its activity in modulating alternative splicing in myoblasts (Lin and Tarn, 2012). The *NCAM1* gene encodes a cell adhesion protein that is a member of cell adhesion molecules (CAMs) family. The CAMs are associated with the binding of a cell to another cell or to the extracellular matrix. They play important role in cell proliferation, differentiation, trafficking, motility, apoptosis and tissue architecture. Up to now, it is not clear how methylation of *RBM4* gene and *NCAM1* gene are involved in DL. Multiple studies have shown that *NCAM1* is associated with denervation and reinnervation, and is often used as a marker of muscle fiber denervation. We cannot definitively know that the *NCAM1* is associated with DL. However, that *NCAM1* is associated with denervation and reinnervation, and is often used as a marker of muscle fiber denervation (Barns et al., 2014; Messi et al., 2016) provides evidence to conclude that *NCAM1* may be involved in WHC of muscle by regulating skeletal muscle fiber.

When consumers choose fresh meat, meat color plays an important visual role. In this study, meat color traits were recorded by a Minolta CR-300 colorimeter. We identified several of significant CpG sites in 2 chromosome regions (SSC12 and SSC16). Yellowness of meat (b*) is mainly influenced by the fat deposits in muscle (Calnan et al., 2017). Usually, yellowness increases with the amount of fat deposited in the muscle. We identified 2 significant CpG sites for b* on SSC12 (Figure S3), which had’n been previously reported. The cpg2272837 is annotated to the upstream of *MED13* gene. The *MED13* is a protein coding gene that encodes a component of the mediator complex. The mediator complex acts as a centralized hub for transcriptional regulation and plays an important role in metabolic control (Amoasii et al., 2018). Previous study reported that *MED13* overexpression enhanced lipid metabolism, insulin sensitivity, and decreased susceptibility to obesity (Baskin et al., 2014). Yellowness of meat is affected by muscle fat deposition. This result could imply that *MED13* may affect yellowness of meat by participating in lipid metabolism. The cpg2270611 is annotated within the *TRIM37* gene which encodes a peroxisomal protein (TRIM37) with E3 ubiquitin-ligase activity. The mutations in the *TRIM37* gene caused mulibrey (muscle-liver-brain-eye) nanism (MUL). Although the physiological function of TRIM37 *in vivo* is unclear, a study has shown that members of the ubiquitin-proteosome pathway can participate in energy metabolism by affecting the regulation of insulin signaling (Karlberg et al., 2005). Energy metabolism plays an important role in the process of muscle transformation after slaughter. Functional studies are needed to investigate the roles of the *MED13* and *TRIM37* gene in meat color among Yorkshire pigs.

A growing body of evidence supports the contribution of epigenetic modification to phenotypic variation in livestock (Triantaphyllopoulos et al., 2016; Wang and Ibeagha-Awemu, 2020) and supports the potential application of epigenetic biomarkers, particularly DNA methylation in livestock breeding programs. Moreover, a previous study has reported the association between SNPs and differential DNA methylation (Imgenberg-Kreuz et al., 2018). This provided one possible mechanism that SNPs impacts gene expression by altering DNA methylation, thereby suggesting the possible application of epigenetic biomarkers in livestock improvement breeding (Maldonado et al., 2019). Thus, in the development of new breeding methods, the relationship between DNA methylation biomarkers identified by EWAS and production traits can be considered in order to be able to quantify the epigenetic contribution to breeding value prediction. (Wang and Ibeagha-Awemu, 2020). Therefore, more studies are needed to get a better understanding of the epigenetic mechanisms underlying phenotyping variation in pig production.

## 4 Materials and methods

### 4.1 Animals and DNA sample

A total of 140 Yorkshire pigs (51 male and 89 female) were used to extracted genomic DNA from muscular tissue. The pigs were raised under the same recommended environment at the conservation farm of Mingxing Agricultural science and Technology Development Co.,Ltd. All individuals were raised to 111.71 Kg ( ±12.97 Kg) weight, transported to the slaughterhouse, and were fasting for 24 h before slaughter determination. After the carcass composition traits were determined, meat quality traits were measured using methods previously described in detail (Duan et al., 2009; Ma et al., 2009). All meat quality measurements were taken on the left side of the carcass. Muscle pH values of the longissimus dorsi muscle were measured at 45 min (pH_45min_) and 24 h (pH_24h_) using a portable pH meter (model 720A; Orion Research Inc., Boston, MA, United States). Meat color, including lightness (L*), redness (a*), and yellowness (b*) were measured at 45 min using a Minolta CR-300 colorimeter (Minolta Camera, Osaka, Japan). Drip loss (DL) was accessed by the method described by Otto et al. (2004). Briefly, a slice of fresh muscle was placed in a plastic bag on a grid parallel to the fiber direction. The weight loss percentages after 24 h of storage at 4°C were calculated. Muscle pH, meat color, and DL were measured in triplicate for each sample, and the average of the three measurements was used. DNA samples from the muscle tissue were snap-frozen in liquid nitrogen then held at −80°C until analysis.

### 4.2 DNA methylation data

Briefly, genomic DNA was isolated from flash frozen muscular tissue. Then, the construction of RRBS libraries and paired-end sequencing using Illumina HisSeq analyzer was performed at Novogene technology co., LTD. (Beijing, China). Raw sequencing data were processed by an Illumina base-calling pipeline. Clean reads were aligned to the pig reference genome (Sscrofa11.1) using Bismark (Felix and Andrews, 2011) after removing adaptor sequences. Next, ML were measured using bismark_methylation_extrator program. A quality control procedure was adopted to ensure the high data quality by 1) retaining only CpG cytosines across all samples; 2) removing CpG sites with missing methylation values at >35 samples; 3) removing CpG sites with coverage <10 reads within a sample. A total of 3,083,713 CpG sites were retained for further analysis. The distribution of CpG sites were conducted using R package Rldeogram v.2.2 (Hao et al., 2020).

### 4.3 Data analysis

We used the R package CpGassoc v2.60 (Barfield et al., 2012) to test for association between methylation and phenotype. We applied the linear mixed-model with the CpG ML score (vary between 0 and 1) as the outcome, and adjusted for sex, year, month, parity and body weight. The following model was used:

y=μ+xβ+u+e

where y is vector of the phenotypes, *μ* is the mean, x is the vector of CpG, β is CpG effect, μ represent random effects, e is the vector of random residuals.

We corrected for multiple comparisons using a Bonferroni correction for 0.05/3,083,713 test setting a significant threshold *p*-value to $7.79\times 10^{-8}$ . We constructed Manhattan plots to present the results of epigenome-wide association analysis using the R package qqman v0.1.8 (Turner, 2018).

### 4.4 Candidate genes and annotation

Then we identified candidate genes based on the significant CpG sites by the Ensemble biomart (http://www.biomart.org). The candidate genes listed for each site correspond to the gene itself for intragenic, and the two nearest flanking genes by distance for intergenic, with the distance between the site and each flanking gene listed for intergenic associations. The gene lists were then submitted for enrichment analysis using the Database for Annotation, Visualization and Integrated Discovery (DAVID) v6.8 (http://david.ncifcrf.gov/). Significant Gene Ontology (GO) terms and Kyoto Encyclopedia of Genes and Genomes (KEGG) pathways were selected after filtering with P<0.05 .

## Data Availability

The data presented in the study are deposited in the Figshare repository, accession number 10.6084/m9.figshare.20633217.

## Supplementary material

Supplementary Figure S1 The Manhattan plot of significant CpG sites on SSC2 for DL trait and the graphic of significant CpG sites in the genomic region. Each dot represents an CpG site and the green dot in the circle represents significant CpG site. The X-axis represents genomic region, and the Y-axis represents average methylation level of CpG sites.

Supplementary Figure S2 The Manhattan plot of significant CpG sites on SSC9 for DL trait and the graphic of significant CpG sites in the genomic region. Each dot represents an CpG site and the green dot in the circle represents significant CpG site. The X-axis represents genomic region, and the Y-axis represents average methylation level of CpG sites.

Supplementary Figure S3 The Manhattan plot of significant CpG sites on SSC12 for b* and the graphic of significant CpG sites in the genomic region. Each dot represents an CpG site and the green dot in the circle represents significant CpG site. The X-axis represents genomic region, and the Y-axis represents average methylation level of CpG sites.

Supplementary Figure S4 The Manhattan plot of significant CpG sites on SSC12 for L* and the graphic of significant CpG sites in the genomic region. Each dot represents an CpG site and the green dot in the circle represents significant CpG site. The X-axis represents genomic region, and the Y-axis represents average methylation level of CpG sites.

Supplementary Figure S5 The Manhattan plot of significant CpG sites on SSC16 for L* and the graphic of significant CpG sites in the genomic region. Each dot represents an CpG site and the green dot in the circle represents significant CpG site. The X-axis represents genomic region, and the Y-axis represents average methylation level of CpG sites.
