# Supplementary figures and images for: Epigenome-wide association studies of meat traits in Chinese Yorkshire pigs highlights several DNA methylation loci and genes
Source: Front Genet. 2023 Jan 4;13:1028711. doi: 10.3389/fgene.2022.1028711 (PMC9845630; doi:10.3389/fgene.2022.1028711)

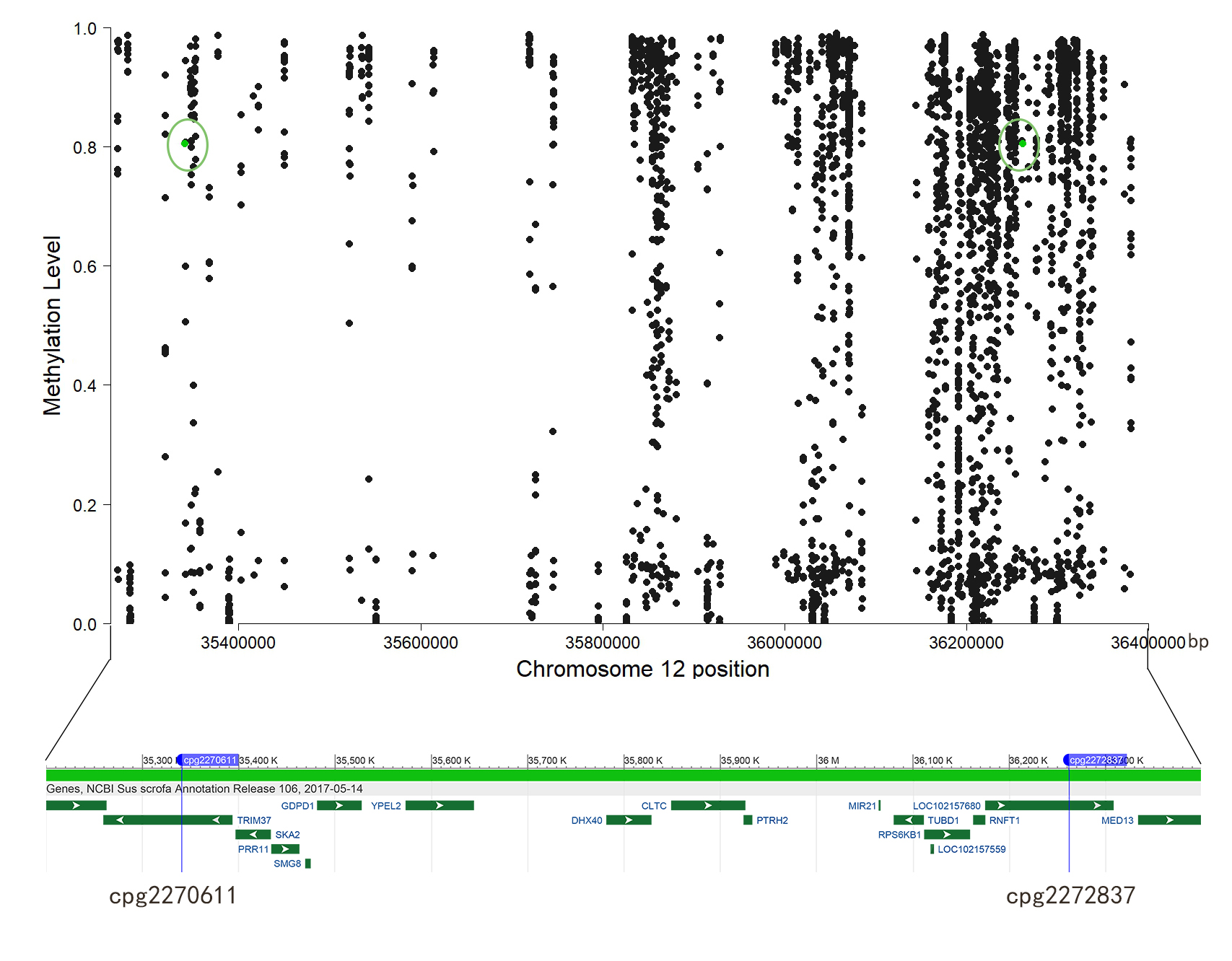

Supplement: Supplementary file 2 [file Image3.JPEG]

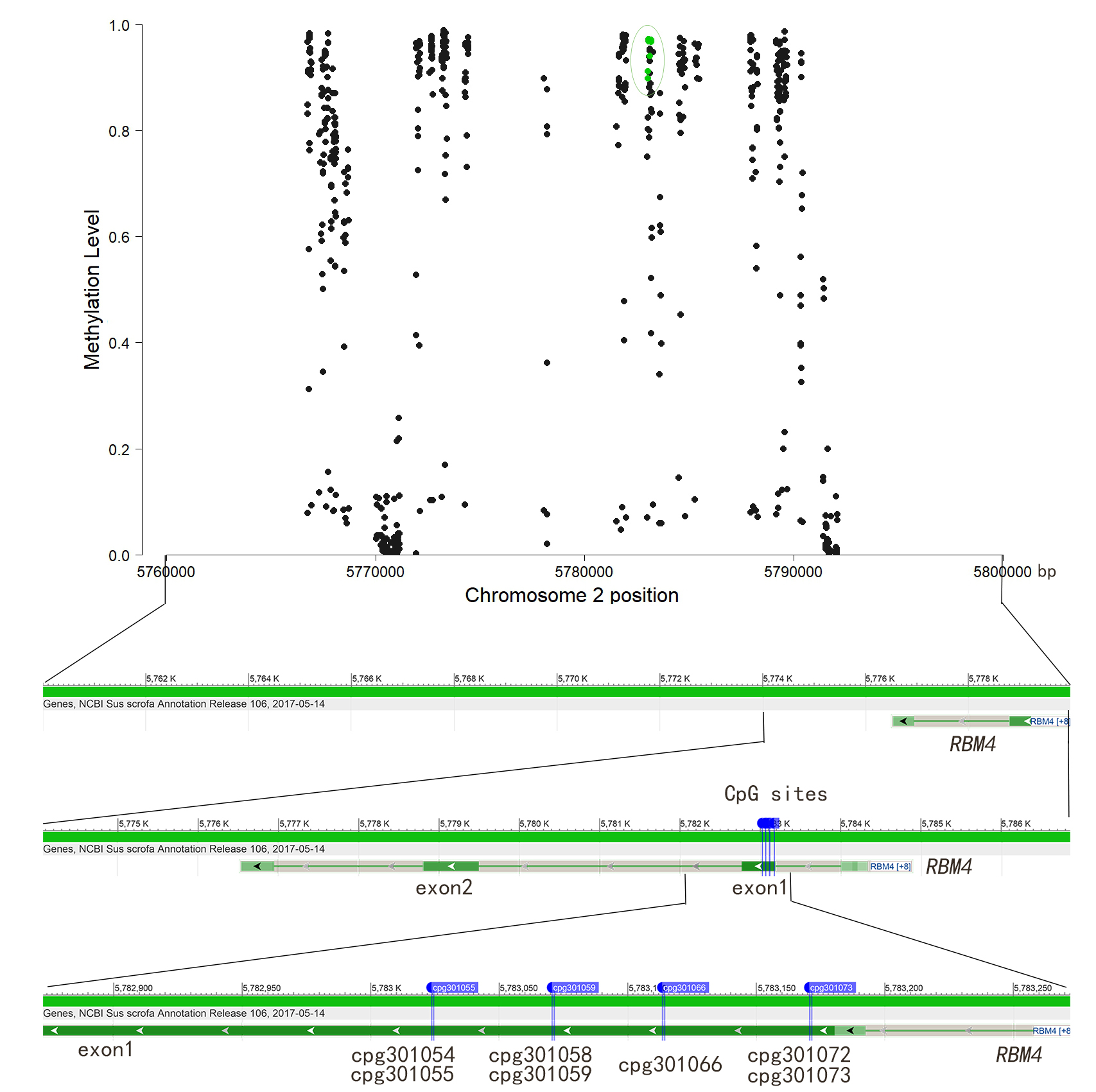

Supplement: Supplementary file 4 [file Image1.JPEG]

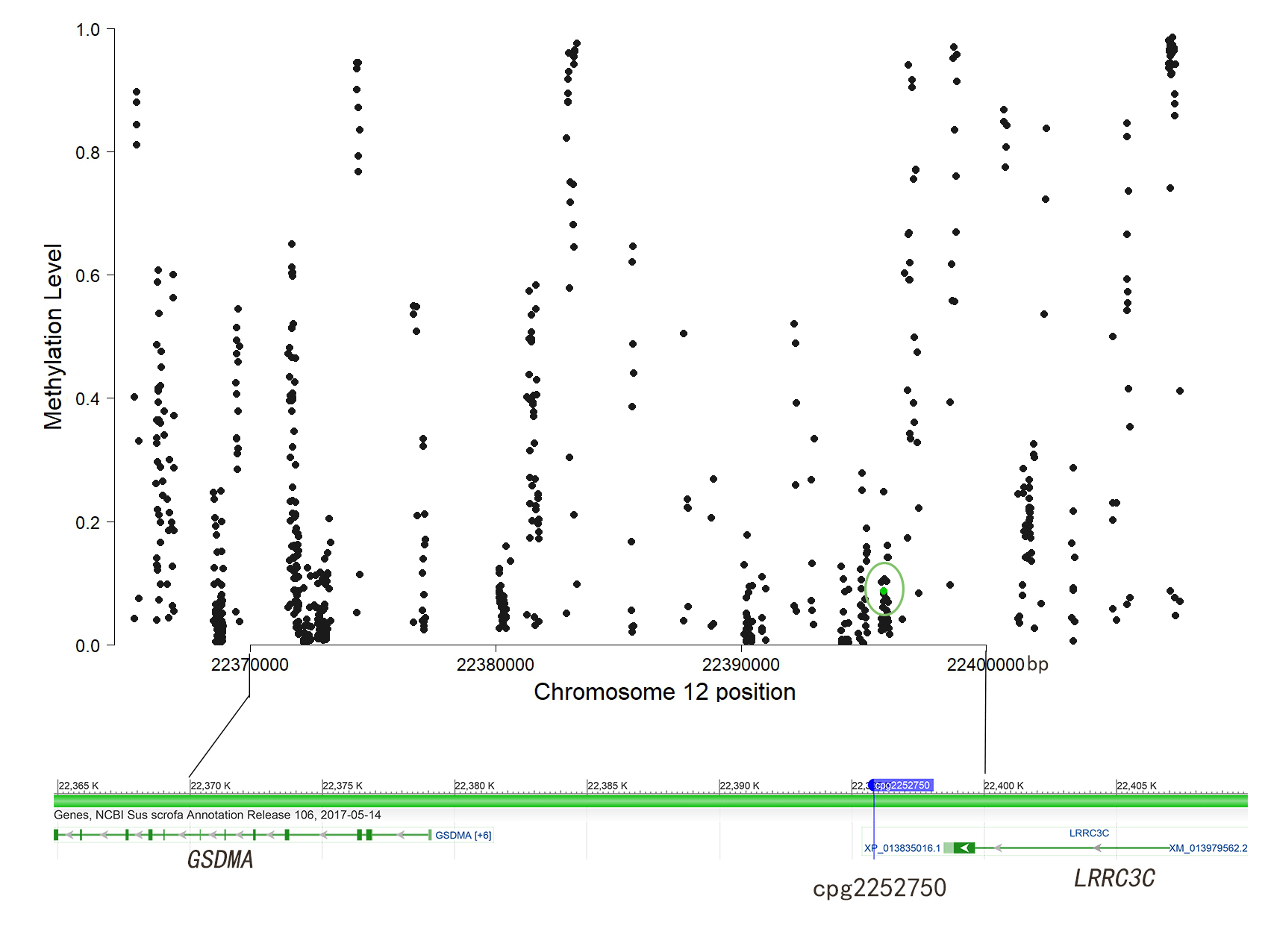

Supplement: Supplementary file 5 [file Image4.JPEG]

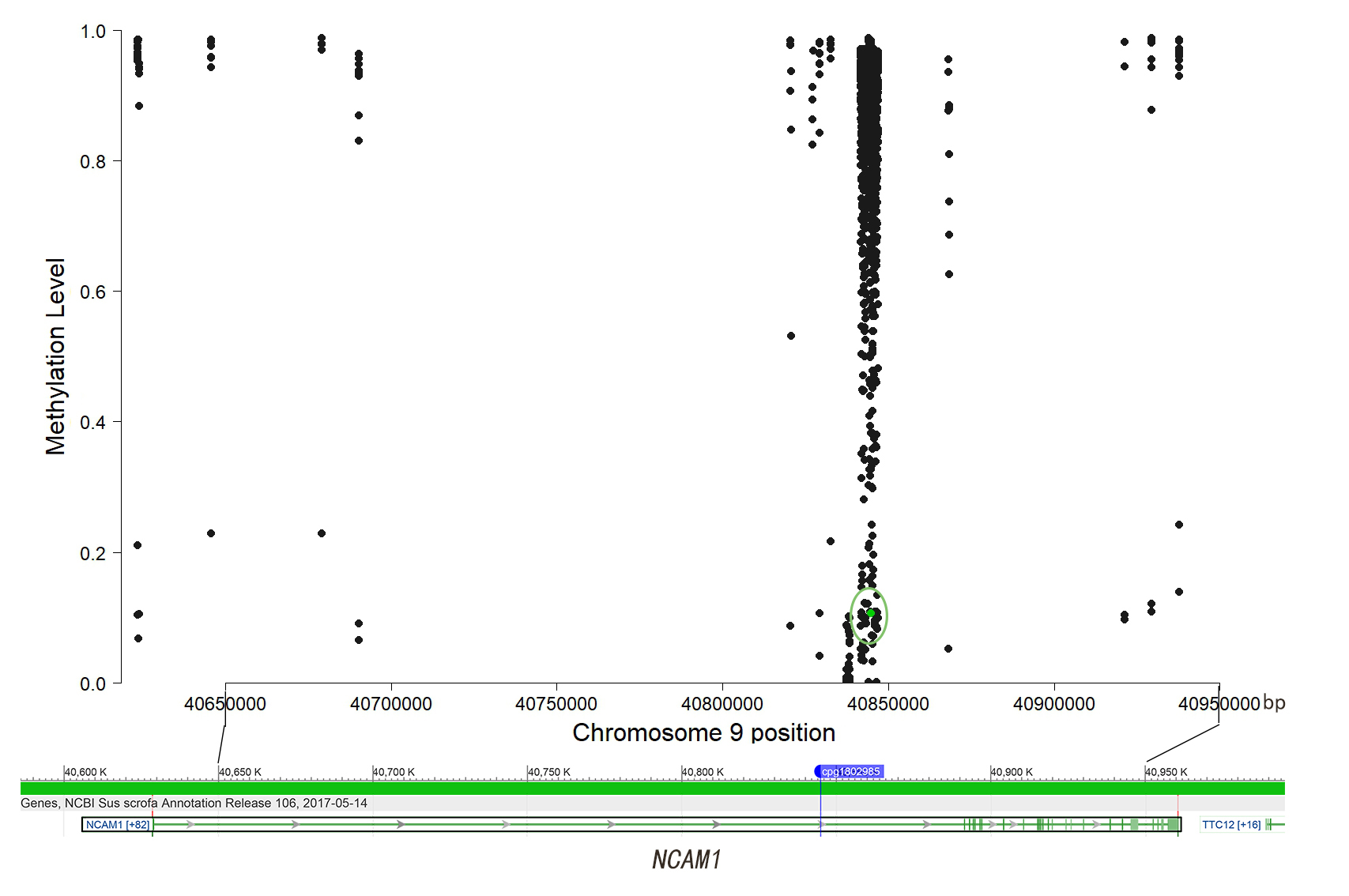

Supplement: Supplementary file 6 [file Image2.JPEG]

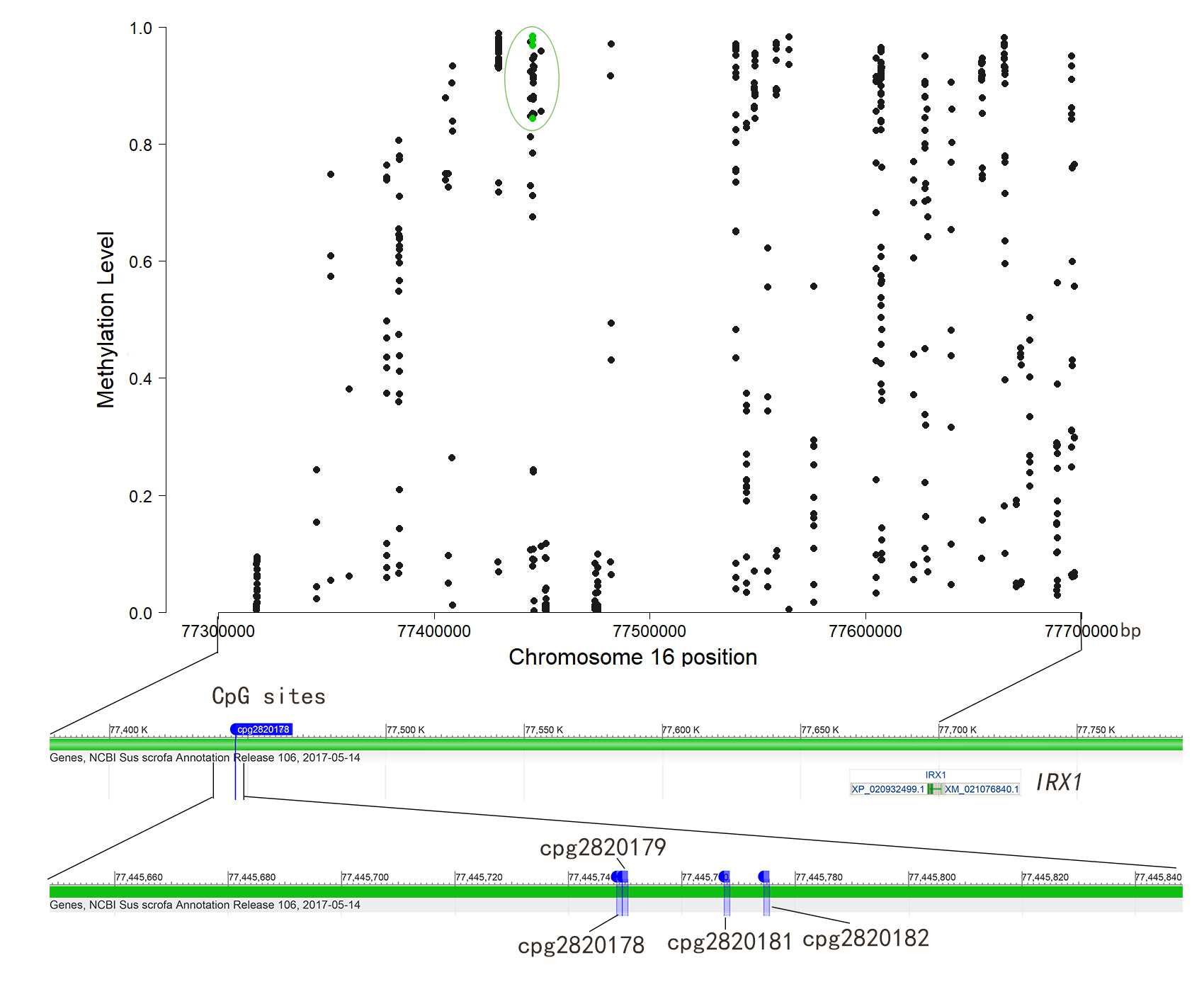

Supplement: Supplementary file 7 [file Image5.JPEG]
